# An Unusual Case of Vulvar Involvement in the Course of Granulomatosis with Polyangiitis

**DOI:** 10.3390/ijerph192113862

**Published:** 2022-10-25

**Authors:** Katarzyna Nowak, Mateusz Kozłowski, Emilia Piekara, Edyta Gołembiewska, Tomasz Huzarski, Aneta Cymbaluk-Płoska

**Affiliations:** 1 Department of Gynecological Surgery and Gynecological Oncology of Adults and Adolescents, Pomeranian Medical University in Szczecin, al. Powstańców Wielkopolskich 72, 70-111 Szczecin, Poland; 2 Department of Nephrology, Transplantology and Internal Medicine, Pomeranian Medical University in Szczecin, al. Powstańców Wielkopolskich 72, 70-111 Szczecin, Poland; 3 International Hereditary Cancer Center, Department of Genetics and Pathology, Pomeranian Medical University in Szczecin, 71-252 Szczecin, Poland

**Keywords:** granulomatosis with polyangiitis, vulva, labium minus, vulvar cancer

## Abstract

Granulomatosis with polyangiitis is a rare autoimmune disease with the presence of c-ANCA in most cases. It involves necrotizing inflammation in small and medium-sized vessels with multiple granulomas. The disease can affect many systems, but the typical triad of attacked systems are the upper and lower respiratory tracts and kidneys, with varying degrees of severity. Involvement of the respiratory tract may manifest, among other symptoms, as nasal crusting, nosebleeds, and dyspnea. Among patients with granulomatosis with polyangiitis, only less than 1% develop genitourinary system involvement. We present a case study of a 36-year-old woman with an 8-year-long GPA history and a lesion, which, due to its appearance and accompanying symptoms, aroused the suspicion of a neoplasm but was proven to be a granuloma with a nontypical location. The systemic disease was treated with glucocorticosteroids and cyclophosphamide. The lesion on the labium minus was surgically removed. We concluded that the macroscopic picture of GPA of the vulva and vulvar cancer is similar. The patient’s medical history may help differentiate GPA and vulvar cancer. Although vulvar GPA is extremely rare, it should be considered in the differential diagnosis of vulvar lesions, especially those suspected to be oncological.

## 1. Introduction

Granulomatosis with polyangiitis (GPA, formerly known as Wegener’s granulomatosis), is a rare autoimmune disease with the presence of c-ANCA (mainly against proteinase 3) in most cases [1]. It is estimated that the incidence of GPA is 4 to 21 people per million per year [2]. Most cases occur between 45 and 60 years although cases are seen in younger people and children [3]. The disease most often affects people of the Caucasian race. GPA occurs with similar frequency in men and women [4]. It involves necrotizing inflammation in small and medium-sized vessels with multiple granulomas [5]. The disease can affect many systems, but the typical triad of attacked systems are the upper and lower respiratory tracts and kidneys, with varying degrees of severity [6]. Involvement of the respiratory tract may manifest, among other symptoms, as nasal crusting, nosebleeds, and dyspnea [7].

As labial involvement is extremely rare, specific guidelines for labial involvement have not been established. Nevertheless, the involvement of the labium as the sole manifestation of the disease or as a co-manifestation can be classified differently. The treatment of GPA distinguishes between treatment induction and maintenance treatment. Glucocorticosteroids (alone or in combination) and methotrexate, cyclophosphamide, rituximab, azathioprine, and mycophenolate mofetil, in combination with glucocorticosteroids, are used [8].

Genitourinary involvement in GPA occurs in less than 1% of cases [9]. In most of the described cases involving genitourinary involvement, gynecologic malignancy was considered the first possible diagnosis [10]. It is worth noting that more frequently than GPA of the vulva, cancer in this area is observed. Squamous cell carcinoma accounts for 90% of vulvar cancers and has an estimated incidence of 2–7 per 100,000 women [11]. Symptoms of vulvar cancer are a lump or visible lesion often accompanied by pruritus, dysuria, or bleeding [12]. As for diagnosis, it is considered that any suspicious lesion on the vulva should be biopsied and evaluated histopathologically [13].

We present a case study of a 36-year-old woman with an 8-year-long GPA history and a lesion which, due to its appearance and accompanying symptoms, aroused the suspicion of a neoplasm but was proven to be a granuloma with a nontypical location.

## 2. Case Report

### 2.1. Gynecological History

**Figure 1:**
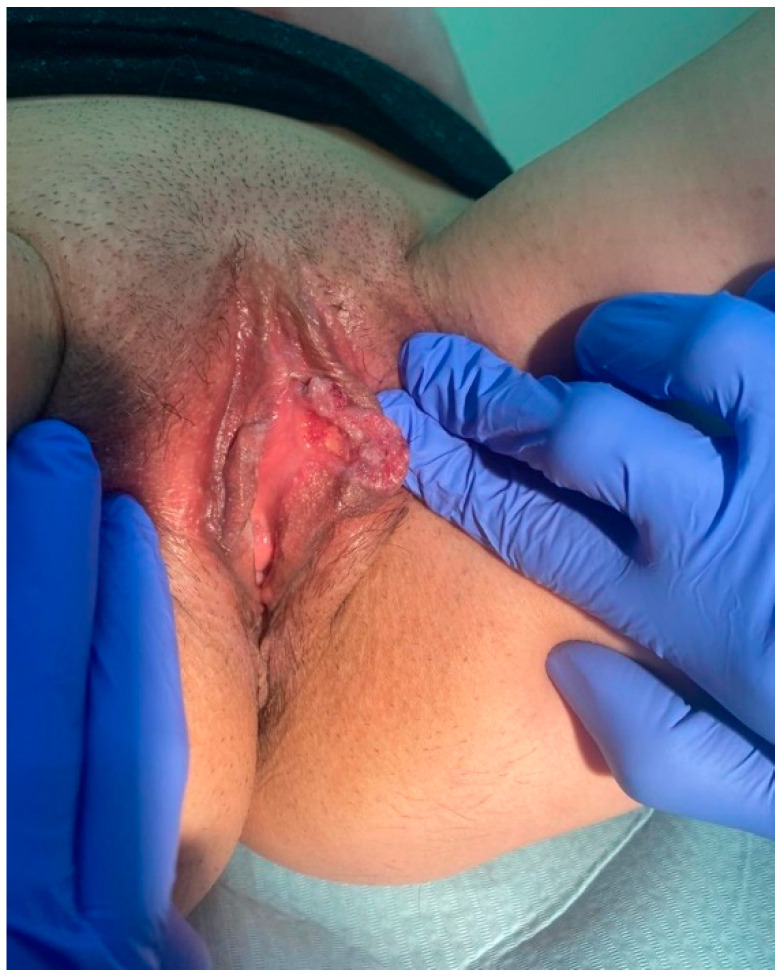
Macroscopic picture of a lesion on the left labium minus.

A 36-year-old Polish woman was admitted to the Department of Gynecological Surgery and Gynecological Oncology of Adults and Adolescents for suspected vulvar cancer. The patient was referred from the gynecology outpatient clinic because of a lesion on the left labium minus. The lesion had been noticed by the patient two months earlier and, from her account, was growing very rapidly. The patient described that the lesion was initially the size of a “pea grain”, two weeks later it had grown approximately four times while on admission it covered over 50% of the labium minus (Figure 1).

The patient reported that the lesion was painful and caused persistent itching. In addition, brownish contents with an unpleasant odor were coming out of the lesion. The presence of the lesion did not affect urination or sexual intercourse, but the patient abandoned these due to psychological discomfort. A month before reporting to the gynecology outpatient clinic, the patient had visited another gynecologist, who performed cytology. The cytology result was correct. The gynecologist also recommended a dermatology consultation. The dermatological treatment that was implemented did not result in clinical improvement, so the patient reported to the gynecological outpatient clinic.

During the outpatient visit, a biopsy of the lesion on the vulva was taken, and the material was submitted for histopathological examination. The result described ulcerated tissue fragments partially covered by squamous multilayered epithelium with focal small-grade intraepithelial neoplasia and a profuse inflammatory infiltrate with a predominance of neutrophils. Features of malignant proliferation were not found. After receiving the biopsy result, the patient was referred to the department. Based on the gynecological examination, transvaginal ultrasound, and in view of the patient’s reported complaints (rapid growth, itching, pain), the lesion raised oncological concerns—vulvar cancer was suspected. Most women with vulvar cancer present to a gynecologist precisely because of a lump or ulcer and the presence of pruritus or pain [14]. Due to this fact, a decision was made to remove the lesion with a margin and submit it for histopathological examination.

### 2.2. Granulomatosis with Polyangiitis

At the age of 28, the patient was hospitalized in the Department of Internal Medicine due to a progressive deterioration of her general condition, shortness of breath, joint pain and swelling, earache, a fever of up to 38 degrees Celsius, a productive cough with a spitting up of a periodic bloody discharge, and a persistent runny nose. The patient’s history included two hospitalizations in the laryngology department for left otitis media. The patient reported periodic nosebleeds. On admission, the patient’s condition was fair; on physical examination, deviations included swelling of the left elbow joint with petechiae, swelling of finger 5 with petechiae on the skin of the proximal metacarpophalangeal joint, swelling of the knee joints, enlarged submandibular lymph nodes, and dry oral mucous membranes. Of the deviations in the laboratory tests, elevated inflammatory parameters and coagulation disorders were noted. The suspicion of sepsis with DIC (disseminated intravascular coagulation) was raised. Due to the patient’s deteriorating general condition despite the implemented treatment, she was transferred to the Intensive Care Unit where she was intubated and mechanically ventilated. After her condition was stabilized, she returned to the Internal Medicine ward. At the aforementioned hospital, diagnostics were performed and from the deviations, elevated inflammatory parameters, anemia, hypoalbuminemia, periodically active urine sediment with proteinuria, elevated D-dimers, and several positive c-ANCA antibodies were found. A chest CT (Computed Tomography) scan showed a lesion 2.2 × 1.6 cm in size at the top of the right lung showing communication with the pleura. In communication with the interlobar fissure of the left lung, a nodule 0.2 cm in diameter was visualized at the border of segment 1/2 and segment 6. Also noted were enlarged lymph nodes of the head and neck, as well as numerous nodes in the neck along the sternoclavicular muscles, 0.5–1.3 cm in size. Virological tests for HIV (human immunodeficiency virus), HBV (hepatitis B virus), and HCV (hepatitis C virus) were negative. Negative microbiological blood and sputum tests (also for tuberculosis) were also obtained. Due to suspected connective tissue disease, the patient had a rheumatology consultation, during which a nasal mucosal specimen was recommended. The result of the specimen examination was inconclusive. Due to the presence of a lung lesion, the patient was transferred to the tuberculosis and lung disease unit. Of the deviations on physical examination, joint edema of variable localization, necrotic skin lesions, and minor swelling of the lower extremities were described. A follow-up CT scan of the chest showed a reduction in the size of the lesion in the right lung with the appearance of new peribronchial lesions. Treatment included antibiotic therapy (doxycycline) with no clinical or radiological improvement. The diagnosis was expanded with bronchofiberoscopy, which revealed lesions in the tracheal mucosa and main bronchi. Based on the histopathological examination of the collected scrapings, tuberculosis was suspected. However, given the reduction in the size of the lung lesion without specific treatment, the repeatedly negative bacteriological results of the sputum and aspirate, and the negative staining of the slide for tuberculosis, this diagnosis was not confirmed. Due to the observation of the patient’s worsening general condition, the appearance of hemoptysis, more severe swelling of the lower extremities and face, and an increase of blood pressure with a concomitant increase in renal parameters, and due to the lack of a definitive exclusion or histopathological confirmation of granulomatosis with polyangiitis with a clinical picture suggestive of this diagnosis, it was decided to refer the patient to the Department of Nephrology, Transplantology and Internal Medicine because of suspected renal involvement. On admission to the department, the patient’s condition was assessed as fair. The patient reported weakness, shortness of breath, a cough, and joint pain; no fever or hemoptysis. On physical examination, from deviations, ulcerations were described in the area of the left elbow joint, left toe, and right hand in the process of healing, and slight swelling of the lower extremities. Laboratory tests from the deviations described normocytic anemia, elevated inflammatory parameters, active urine sediment, positive c-ANCA antibodies, anti-proteinase-3 (PR-3) antibodies, and negative anti-myeloperoxidase (MPO) antibodies. During hospitalization, a renal biopsy was taken—the histological picture was consistent with the diagnosis of vasculitic glomerulonephritis. Remission-inducing treatment with glucocorticosteroids and cyclophosphamide was started. The treatment included pulses of methylprednisolone (6 × 500 mg intravenously), followed by oral prednisone and the first pulse of cyclophosphamide (500 mg intravenously). Omeprazole at a dose of 20 mg once daily was also included in the treatment. For the next 5 months, the patient reported to the department approximately every month for the administration of further pulses of cyclophosphamide (6 pulses in total). At that time, the patient was diagnosed with hypertension and its treatment was started. During the third hospitalization in the department, the patient complained of shortness of breath, cough, and facial swelling. The patient was consulted in the Department of Otolaryngology. From the significant deviations of a videolaryngoscopy, stenosis of the subglottic region was described, with thick, obstructive secretions in the tracheal lumen. A mucolytic treatment, inhaled steroid therapy, and antireflux treatment (increasing the dose of omeprazole to 40 mg/day) were recommended, as well as a follow-up videolaryngoscopy after two months, and if there was no improvement, a CT scan of the larynx with the assessment of subglottic stenosis. On admission to the fourth pulse, the patient complained of shortness of breath, facial edema, hirsutism, and weight gain. After the administration of cyclophosphamide, it was decided that the next two pulses would be at a dose of 1000 mg. Due to the side effects of prednisone, a switch from this medication to deflazacort was made. Due to persistent proteinuria, enalapril at a dose of 2 × 5 mg was included in the treatment. During a subsequent hospitalization, enalapril was discontinued due to worsening dyspnea and a persistent cough. During the hospitalization, during which the last pulse of cyclophosphamide was given, the patient complained of exertional dyspnea, facial edema, and hirsutism. In addition, the patient was consulted by a laryngologist. The consultation revealed that the laryngeal stenosis had decreased.

The patient then started maintenance therapy with glucocorticosteroids and was transferred to the care of Nephrology Outpatient Care where the patient had regular visits every few months. Four years after the last pulse of cyclophosphamide, progressive proteinuria without hematuria began to be observed. As a result, the patient was again hospitalized in the Department of Nephrology, Transplantology, and Internal Medicine for observation for a recurrence. On admission, the patient reported no complaints, and no significant abnormalities were described on physical examination. Laboratory tests of abnormalities described subrenal proteinuria and a slightly elevated creatinine level. Due to increasing proteinuria, the dose of deflazacort was increased to 12 mg per day. Hospitalization for another month was set. During the subsequent hospitalization, based on the results of the tests performed (negative determination of ANCA antibodies, daily proteinuria of less than 1 g), it was recommended to maintain the current treatment, and the patient was referred for further care in the outpatient clinic. Currently, the patient is under the further care of the Nephrology Outpatient Clinic.

### 2.3. Histopathological Examination of the Lesion on the Labium Minus

**Figure 2:**
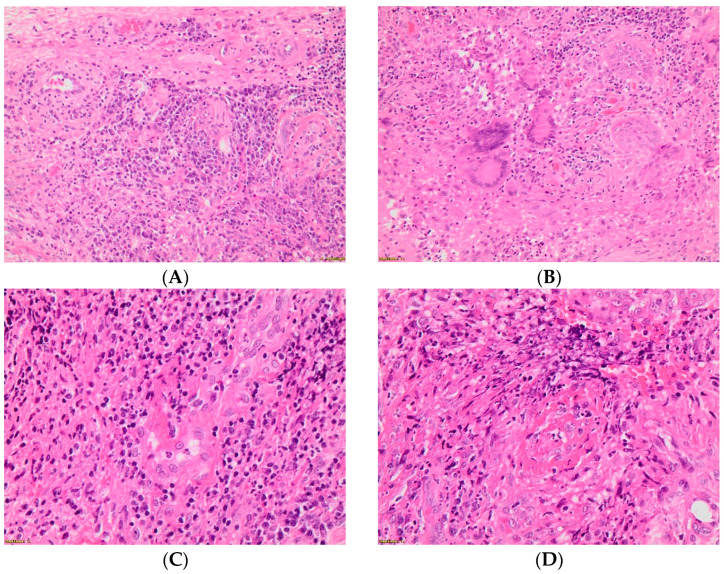
(**A**–**D**) Histological image of granulomatosis with polyangiitis of the vulva in the described patient.

The lesion on the labium minus was surgically removed. After removal of the lesion from the labium minus two tissue fragments were submitted for histopathological examination. Sections were taken from both and analyzed. The result showed that the morphological features may correspond to Wegener’s granuloma-type lesions. Neoplastic proliferation was not found (Figure 2). Immunohistochemistry: P16(−), panCK(+), CD68(+), Ki-67(+). Due to this result, vulvar cancer was excluded and the lesion was found to be related to granulomatosis with polyangiitis.

### 2.4. Further Medical Care

Four months after hospitalization in the Department of Gynecological Surgery and Gynecological Oncology of Adults and Adolescents, the patient felt the onset of a lesion similar to the one described above on the right labium. Accordingly, the patient was hospitalized in the Department of Internal Medicine, Rheumatology, Diabetology, Geriatrics, and Clinical Immunology for further management. During the hospitalization, a gynecological consultation was held, in which it was recommended that if there was no effect of the treatment of the underlying disease, the growth of the labium minus lesion, and complaints of pain, the patient should undergo elective surgery to remove the lesion. Based on the overall clinical picture and the results of the tests performed, it was decided to treat the patient with pulses of methylprednisolone (3 times 500 mg intravenously) and cyclophosphamide (800 mg intravenously). It was recommended to report for another pulse of cyclophosphamide in the following month.

## 3. Discussion

GPA is a rare autoimmune disease and it is classified as one of the antineutrophil cytoplasmic antibody (ANCA)-associated vasculitides [5]. It can affect numerous systems, the respiratory and urinary in particular. The occurrence of upper respiratory tract manifestations is estimated at 95%, with the nasal cavity and sinuses as the most common areas of involvement [15]. Patients report nasal hemorrhages, ulcerated mucosa in the nose and oral cavity, and sinusitis. Often, recurrent otitis can be the first symptom. Approximately half of the outpatients develop otological abnormalities early [16]. Lung manifestations come, among others, in the form of dyspnea, cough, pulmonary hemorrhage, respiratory failure, or pulmonary nodules that need to be differentiated from neoplasma or tuberculosis. As for renal involvement, glomerulonephritis is observed on renal biopsy and erythrocytes are present in urine analysis. In addition to respiratory and renal involvement, GPA often manifests with symptoms in the eyes, joints, or nervous system. It may also affect the skin, and cardiovascular or gastrointestinal systems [4]. In our case, the patient presented to the hospital with symptoms of respiratory involvement such as a cough, shortness of breath, and expectoration of bloody sputum. In addition, joint swelling was observed, and the patient reported joint soreness. The patient had a history of two otitis media and reported periodic nosebleeds. Of the abnormalities characteristic of GPA, periodic active urine sediment and an elevated c-ANCA titer were observed in the test results. During further diagnosis and treatment, the patient was diagnosed with a lung lesion, and vasculitic glomerulonephritis was observed in a kidney biopsy.

Among patients with granulomatosis with polyangiitis, only less than 1% develop genitourinary system involvement [17]. To the best of our knowledge, only a few cases of GPA with vulvar involvement have been described. Lewis described the case of a 54-year-old woman who had lesions on both labia minora. Symptoms reported by the patient included vulvar soreness and contact bleeding [18]. Our patient also reported pain, but the main complaint she gave was persistent itching. Swain et al. described the case of an 8-year-old girl who developed a granuloma within the left labium majus in the course of another vasculitis—Eosinophilic Granulomatosis with Polyangiitis (formerly Churg-Strauss syndrome). The diagnosis of the lesion in the labium majus, as in our patient, was based on a biopsy of the mass [19]. As for the involvement of other genital organs, there have been a few more cases described. The authors describe lesions associated with granulomatosis with polyangiitis in female patients in the urethra, vagina, uterus, cervix uteri, or ovaries [17,20,21,22,23,24,25,26,27,28,29]. Because vulvar lesions in the course of GPA are so rare, there are no diagnostic or treatment standards. In our case, both surgical and pharmacological treatment of the vulvar lesion was applied, while in the case described above of a 54-year-old patient with involvement of both labia minora, the woman was treated with systemic prednisone followed by prednisone with azathioprine. Originally, our patient had multiple organ involvement. Vasculitic glomerulonephritis was also diagnosed. Such a disease should be classified as active severe. The patient was then started on a systemic treatment: glucocorticosteroids and cyclophosphamide. The patient was put into remission, which was maintained with glucocorticosteroid treatment. Subsequently, the involvement of the left labium occurred. The manifestation of the primary disease in the form of a lesion on the labium may be classified as an active nonsevere disease. However, the patient should be diagnosed with a relapse. Due to the uncertain clinical presentation of the lesion and the suspicion of vulvar cancer, the lesion was first biopsied and then surgically removed. Four months later, the patient developed a macroscopically similar lesion on the right labium, which can be classified as a relapse with active nonsevere manifestation. Recommendations of the American College of Rheumatology/Vasculitis Foundation include treatment regimens for patients with GPA who have experienced a relapse with severe disease manifestations [8]. There are no regimens for patients with GPA who have experienced a relapse with nonsevere disease manifestations. As the patient had been previously put into remission with cyclophosphamide and glucocorticosteroids, this treatment was also implemented in this case and is still being maintained. In our opinion, with the current state of knowledge, it is difficult to assess whether surgical or systemic treatment is more appropriate. Moreover, although GPA-induced vulvar lesions are so rare, they should be kept in mind in the differential diagnosis of vulvar tumors and ulcers, especially in patients with a history of GPA.

## 4. Conclusions

GPA is a disease involving multiple organs and systems and requires the cooperation of multiple specialists. The macroscopic picture of GPA of the vulva and vulvar cancer is similar. The patient’s medical history may help differentiate GPA and vulvar cancer. Although vulvar GPA is extremely rare, it should be considered in the differential diagnosis of vulvar lesions.

## Data Availability

Not applicable.
